# Alternatives to Antibiotics in Semen Extenders: A Review

**DOI:** 10.3390/pathogens3040934

**Published:** 2014-12-15

**Authors:** Jane M. Morrell, Margareta Wallgren

**Affiliations:** 1 Clinical Sciences, Swedish University of Agricultural Sciences (SLU), Box 7054, SE-75007 Uppsala, Sweden; E-Mail: margareta.wallgren@slu.se; 2 Quality Genetics Sweden HB, SE-24292 Hörby, Sweden

**Keywords:** antibiotics, semen extenders, resistance, alternatives

## Abstract

Antibiotics are added to semen extenders to be used for artificial insemination (AI) in livestock breeding to control bacterial contamination in semen arising during collection and processing. The antibiotics to be added and their concentrations for semen for international trade are specified by government directives. Since the animal production industry uses large quantities of semen for artificial insemination, large amounts of antibiotics are currently used in semen extenders. Possible alternatives to antibiotics are discussed, including physical removal of the bacteria during semen processing, as well as the development of novel antimicrobials. Colloid centrifugation, particularly Single Layer Centrifugation, when carried out with a strict aseptic technique, offers a feasible method for reducing bacterial contamination in semen and is a practical method for semen processing laboratories to adopt. However, none of these alternatives to antibiotics should replace strict attention to hygiene during semen collection and handling.

## 1. Introduction

One of the benefits of artificial insemination (AI) lies in reducing the spread of disease by avoiding contact between, or transport of, animals for breeding. Paradoxically, AI is one of the ways in which pathogens can be spread easily [1], and therefore, males are tested before entering a breeding program and at several times during the period of semen collection. However, it is impractical to test each ejaculate before insemination. Contaminated semen or semen from infected individuals represents a source of potential disease transmission to susceptible females. Thus, the inclusion of antimicrobials in semen extenders is required by both national and international regulations to control the microbial content. Although frozen semen retains its microbiological profile during storage, it is susceptible to deterioration on thawing; fresh and cooled semen is likely to deteriorate during storage and transport.

Most ejaculates collected from healthy animals are contaminated with bacteria to some extent, e.g., ejaculates from boars [2], stallions [3] and bulls [4]. Further contamination may occur during semen processing [5], which usually takes place without access to a laminar air flow hood. The addition of antibiotics to semen extenders to control the growth of these contaminants is stipulated by national and international guidelines. Since antibiotics may be toxic to spermatozoa, a cocktail of agents is used in semen extenders to reduce the effect of each individual component. Gentamicin, a commonly-used antibiotic in commercial semen extenders, was shown to have an adverse effect on sperm motility and viability [6]. However, there are anecdotal accounts that bacteria in some European countries are resistant to the stipulated antibiotics; hence, further antibiotics are added to control resistant bacteria. Although many countries are trying to reduce the general use of antibiotics to decrease the development of resistance, the addition of antibiotics to semen extenders represents an increasing use, which could be avoided or better regulated if further knowledge is available. Antibiotics in semen extenders are of particular concern in the pig and horse breeding industries, which rely mostly on the use of liquid semen rather than frozen semen.

Bacteria have a negative effect on sperm quality, either by directly competing with spermatozoa for nutrients supplied by the semen extender or by the production of toxic metabolic byproducts and endotoxins. Moreover, bacteria may cause inflammation or disease in inseminated females. The female reproductive tract is exposed to bacterial contamination from the male during natural mating and has therefore developed natural defense mechanisms to remove bacteria. However, when AI is used, the semen may be deposited in a non-physiological part of the female reproductive tract, e.g., bull spermatozoa are deposited in the anterior cervix or uterine body during AI instead of in the vagina, and the deposition of semen must occur at the correct time. Moreover, the semen may have been stored in the presence of a nutrient-rich extender that favors the growth of microorganisms. Therefore, antibacterial agents are added to the semen extenders to prevent disease.

Government and regional directives stipulate which antibiotics, and what doses, should be added to semen doses for international trade. Since food-producing animals, as well as smaller numbers of companion animals (horses and dogs) are currently bred by AI, very large volumes of semen extenders containing antibiotics are used in the animal breeding industry. The use of antimicrobials is not without problems: many have a detrimental effect on sperm survival, and the choice of agents that can be added to semen extenders is limited. There is a tendency to use a cocktail of broad spectrum, highly potent antibacterials, each at a lower concentration than would be required individually in an effort to reduce sperm toxicity. However, even a small amount of antibiotic usage can result in the development of antibiotic resistance within an animal species, for example tetracycline-resistant strains of *Clostridium perfringens* in broiler chickens [7]. Therefore, it would be prudent to reduce antibiotic usage as much as possible. Finding alternatives to antibiotics to control microorganisms in semen doses for AI would be beneficial both to improve sperm quality and survival and to slow the development of antibiotic resistance.

## 2. Specific Effects of Bacteria on Sperm Quality

The presence of contaminant bacteria in extended boar semen is associated with a decrease in sperm motility and viability [8,9], premature acrosome reaction [10] or sperm agglutination [11]. Furthermore, bacteria may cause the production of antibodies directed against the sperm glycocalyx complex [12,13]. The insemination of contaminated semen may be associated with vulvar discharge and returns to estrus [14], or embryonic or fetal death, endometritis, systemic infection and/or disease in recipient females [15], or reduced litter size [16]. However, these effects may depend on the bacteria concerned, since Pasing* et al.* [17] did not detect any adverse effect of the microbial flora on sperm quality in stallions. The microbial flora in their study consisted predominantly of coagulase-negative staphylococci, alpha-hemolytic streptococci and coryneforms, with few potentially pathogenic agents, such as *E. coli* or *P. aeruginosa*, being found. Alternatively, the lower storage temperature of stallion semen compared to boar semen (6 °C and 16–18 °C, respectively) may have contributed to the lack of deleterious effects on stallion semen.

Some attempts to quantify the effect of bacteria on sperm quality have used “spiked” samples,* i.e.*, where known doses of cultured bacteria were added to the semen samples, which were then stored under conventional storage conditions. Haines *et al.* [18] and Sepúlveda *et al.* [19] were able to show deteriorating sperm quality in terms of motility and membrane integrity in poultry semen and boar semen, respectively, associated with increasing bacterial loads.

## 3. Antibiotics Added to Semen Extenders

According to European Council Directive 90/429/EEC, Annex C2 [20], “An effective combination of antibiotics, in particular against leptospires and mycoplasmas, must be added to the semen after final dilution”. This combination must produce an effect at least equivalent to the following dilutions:

- 500 µg per mL streptomycin final dilution;
- 500 IU per mL penicillin final dilution;
- 150 µg per mL lincomycin final dilution;
- 300 µg per mL spectinomycin final dilution.

Figures for the number of AIs carried out in farm animals are difficult to find because of differences in the way in which such data are recorded, e.g., as the number of first services, the number of breeding females,* etc.* However, Thibier and Guerin [21] reported that in 1999, 264 × 10^6^ bull semen doses were produced worldwide. This represents 66,000 L of antibiotic-containing extender, if all of the semen doses consisted of 0.25 mL extended semen containing antibiotics. This calculation does not take into account extender prepared, but not used, or semen doses discarded. Morrell and Wallgren [22] calculated that 4 × 10^6^ L of boar semen extender containing antibiotics were used in the European Union annually, based on a population of breeding sows of approximately 14 million, each of which is expected to produce 2.3 litters of piglets per year. The European Union is not the world’s largest pig producer; thus the potential global usage of antibiotic-containing extenders for boar semen is quite staggering.

## 4. Presence of Microorganisms in Semen Despite the Addition of Antibiotics to Semen Extenders

Microorganisms were detected by MALDI-MS in 11 out of 30 samples of commercial frozen bull semen doses containing unspecified antibiotics [23]. The organisms were identified as most probably *Citrobacter freundii*, 2/11, *Enterobacter* spp., 5/11 (e.g., kobei, asburiae, hormaechei), *Stenotrophomonas maltophilia*, 2/11, *Enterococcus faecium*, 1/11, and *Candida parapsilosis*, 1/11. In contrast, Guimaraes *et al.* [24] cultured at least 15 species of microorganisms in 20 frozen thawed stallion semen doses containing amikacin; the prevalence of these organisms is shown in Table 1. Furthermore, gentamicin, tylosin, spectinomucin and lincomycin did not control bacterial growth in bull semen, whereas no growth occurred in samples containing ceftiofur/tylosin or ofloxacin in the extender [25].

**Table 1:** Prevalence of microorganisms isolated from stallion semen samples extended in a cryomedium containing amikacin (*n* =20).

Organisms	Prevalence
*Enterococcus* spp.	100%
*Staphylococcus coagulase negative*	70%
*Yeasts*	65%
*Bacillus* spp.	60%
*Corynebacterium* spp.	50%
*Aspergillus* spp.	20%
*Streptococcus* spp.	5%
*Aerococcus* spp.	5%
*Propionibacterium* spp.	5%
*Micrococcus* spp.	5%
*Bacteroides* spp.	5%
*Rhodococcus* spp.	5%
*Dermobacter* spp.	5%
*Actinomyces* spp.	5%
*Microbacterium* spp.	5%

Note: summarized from [24].

These recent accounts by Zampieri *et al.* [23], Guimaraes *et al*. [24] and Gloria *et al.* [25] indicate that some microorganisms are currently resistant to the antibiotics added to semen extenders for domestic livestock. They may have an effect on sperm quality, since the presence of microrganisms was negatively related to sperm quality in some of the stallion semen samples studied [24]. Similar studies have not been reported for commercial boar semen doses, although there are anecdotal accounts of resistant bacteria appearing in boar semen in some European countries.

## 5. Reducing Contamination of Semen during Collection

Strict attention to hygiene can reduce bacterial contamination during semen collection [5]. The normal flora of the skin, hair and respiratory tract of the male cannot be reduced, although the collector can minimize his/her own contribution through personal hygiene and the sterility of the collection equipment. Protective clothing and footwear, laundered regularly or disinfected as appropriate, should always be worn. People with respiratory infections should avoid collecting semen or, if this is too restrictive, they should wear disposable masks. The semen collection area and equipment should be thoroughly cleaned and disinfected. However, since soaps and disinfectants are spermicidal to some degree, residues of these substances should be avoided on surfaces that will be in contact with semen. All semen collection stations have procedures regarding cleanliness and hygiene, which should be strictly followed at all times. The possibility of contaminating sperm samples by exposure to bacteria in the laboratory environment can be seen from the article by Morrell* et al.* [26], where contamination of a previously unopened bottle of semen extender with 2 × 10^3^ cfu/mL *E. coli* occurred by opening the bottle without access to a laminar air flow bench.

## 6. Alternatives to Conventional Antibiotics in Semen Extenders

Despite the urgent need to find alternatives to conventional antibiotics for use in semen extenders, reports on potential novel antimicrobials have been scarce. The exception is that cationic antimicrobial peptides have been investigated as a novel additive for boar semen extenders by Schulze* et al.* [27]. These peptides may act through destabilization of the bacterial cell membrane, which should mean that it is not possible for resistance to develop. Schulze* et al.* [27] found that low concentrations of some cyclic hexapeptides (known as c-WFW and c-WWW) gave comparable results to gentamycin (a widely-used antimicrobial for semen extenders) in terms of the lack of effect on sperm quality or on pregnancy rate following AI. The peptide c-WFW in particular had a beneficial effect on sperm motility. However, not all members of this class of compounds were suitable for use in semen extenders, since a synthetic magainin II amide derivative, MK5E, was associated with decreased sperm motility and decreased membrane integrity accompanied by irreversible mobility of phosphatidylcholine inthe sperm membrane.

## 7. Physical Removal of Bacteria from the Ejaculate

Several groups have reported the use of colloid centrifugation of semen as a means of physically separating spermatozoa from bacteria in the ejaculate [28,29]. Human spermatozoa were prepared by density gradient centrifugation (DGC) [28], whereas boar spermatozoa were prepared by the simpler method of Single Layer Centrifugation (SLC) [29]. In the latter study, boar spermatozoa survived longer than unselected controls, at room temperature (20–22 °C) and without antibiotics in the semen extender. These results led to the speculation that sperm survival is prolonged in an environment that is free of both bacteria and dead or dying spermatozoa.

Colloid centrifugation of semen, reviewed by Morrell [30], is a relatively simple procedure that is well within the capabilities of most semen processing laboratories. In the original DGC method, the colloid formulation with the highest density (2 mL) was poured into the centrifuge tube and colloids of lesser density (each of 2 mL) were pipetted on top, taking care not to mix the different layers. Up to 1.5 mL of semen were pipetted on top of the colloid, again taking care not to mix the layers. The preparation was centrifuged at 300× *g* for 20 min, before aspirating the supernatant and almost all of the colloid. The sperm pellet was then harvested from beneath the remaining colloid using a sterile pipette and transferred to a clean tube containing sterile extender.

**Figure 1:**
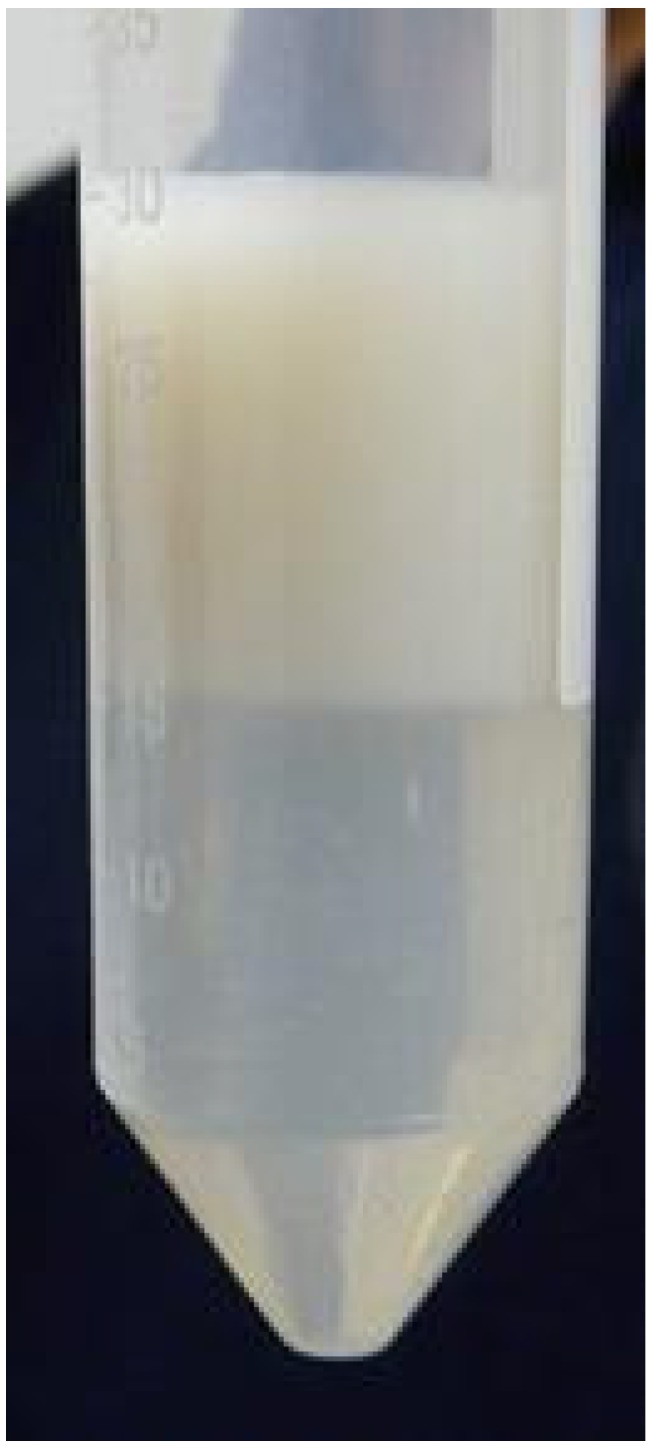
Single-layer centrifugation of stallion semen using Androcoll-E.

Note: the 50-mL centrifuge tube contains 15 mL of extended stallion semen (at a sperm concentration of not more than 100 × 10^6^/mL) carefully pipetted on top of the ready-to-use colloid formulation, Androcoll-E.

For SLC, only one layer of colloid is used, thus simplifying the procedure of preparing the sample. The ready-to-use colloid is poured into a centrifuge tube, and extended semen is layered carefully on top (Figure 1). For optimal sperm selection, the sperm concentration should not exceed approximately 100 million per mL [31] to avoid overloading the colloid. Centrifugation and retrieval of the sperm pellet are done as described for DGC.

Apart from the saving in time in the preparation, the SLC procedure can be scaled-up to enable much larger volumes of semen to be prepared than are possible with DGC. Thus, instead of 1.5 mL of semen on top of 4 mL colloid (2 mL per layer) in a 10-mL tube for DGC, it is possible to prepare different volumes according to the volume of the ejaculate, e.g., 18 mL semen on 15 mL colloid for stallion semen [32], up to 150 mL of extended semen on top of 150 mL colloid in a 500 mL tube with SLC [33]. With the access to disposable pipettes, a pipette-filler for layering the semen and a vacuum pump for removing the supernatant, a skilled operator can prepare the extended sperm-rich fraction of a boar ejaculate (roughly 450 mL) in approximately 30 min, including the 20-min centrifugation.

## 8. Removal of Bacteria by Colloid Centrifugation of Human, Boar and Stallion Semen

In a study with human semen samples processed by DGC using a silane-coated silica colloid, a ten-fold reduction in bacterial count was achieved, and no bacterial growth was observed in 59% of the 68 samples after DGC [28]. Bacterial contamination was almost abolished if strict aseptic techniques were used, changing pipette tips and tubes. The authors considered that the level of* g* force for centrifugation did not affect the removal of bacteria. However, although the sample size was small, it seems that there might have been an increase in the number of positive samples for some bacteria with increasing *g* force, although the results reported in the different tables appear to be conflicting [28]. The types of bacteria isolated were coagulase-negative staphylococci, α-hemolytic *Streptococcus*, Group B *Streptococcus*, *Enterococcus*, *Escherichia coli*, *Proteus* sp*.*, Gram-negative rods (non-lactose fermenting roads) and *Bacillus* sp.

For 10 boar semen samples processed by SLC, where the sperm pellets after SLC were harvested in a laminar air flow (LAF) bench, no bacteria could be cultured from six of the SLC-selected sperm samples, while numbers of bacteria were reduced compared to the controls (uncentrifuged samples) in the remaining cases [29]. The bacterial loads remaining after SLC are shown in Table 2. If the results from two of the SLC samples (which were likely re-contaminated after SLC) are excluded, less than 1% of bacteria remained after the SLC processing. The close similarities with the DGC study on human semen described previously are interesting considering that the starting contamination would be expected to be much higher for boar semen due to the environmental conditions during semen collection. However, in contrast to the previous study with human semen, where no bacteriostatic effect of the colloid was observed, a possible slowing in the rate of bacterial multiplication was seen in the study with boar semen using Androcoll-P, despite the fact that no antibiotics were included in this colloid formulation. Some differences between bacteria were observed in the ease with which they could be removed by SLC, with a tendency for flagellated (and therefore, motile) bacteria to pellet with the spermatozoa. The type of bacteria and/or the length of time between collection and SLC-processing also affected removal.

**Table 2:** Proportion of bacteria remaining in boar sperm samples after processing by single-layer (SLC) centrifugation through Androcoll-P (*n* = 10).

Boar	Bacterial Load at 0 h after SLC (%)	Bacterial Load at 24 h after SLC (%)
1	0	0
2	200 *	0
3	0	0
4	0	0
5	0	0
6	0	0
7	5	2
8	5	6
9	83 *	12
10	27	30
total	<1	<1

Note: There was possible re-contamination of sperm pellet after SLC, because the aliquot taken from the same SLC sample after 24 h had a much lower or no bacterial load. In addition, the contaminating bacteria were isolated in pure culture, in contrast to the original sample. Summarized from [29].

Guimaraes *et al.* [24] performed SLC on stallion semen, although using a different centrifugation protocol to that described for the previously-mentioned studies on human and boar semen. Their SLC preparations were subjected to a centrifugation at 600× *g* for 10 min, and the resulting sperm pellet was resuspended in antibiotic-containing semen extender. They removed on average approximately 50% of the contaminating bacterial load. However, post-thaw sperm motility was increased in samples showing a reduction in bacterial load compared to the controls, confirming the beneficial effects of removing bacteria on sperm quality and survival [24]. It is somewhat surprising that so many bacteria were cultured from the SLC sperm samples in extender-containing antibiotics, indicating a potentially high level of antibiotic resistance, whether the bacteria passed through the colloid during centrifugation at the higher *g* force or whether they were the result of post-centrifugation re-contamination. Certainly, a greater variety of organisms (14 types of bacteria, plus yeasts) were isolated from the stallion semen in Portugal than the nine types isolated from a study at the Gluck Equine Research Center in Kentucky, USA [34], described in the next section.

## 9. Modified Colloid Centrifugation (with a Tube Insert)

One of the difficulties with colloid centrifugation, particularly when working with density gradients, is re-contaminating the sperm pellet during extraction from the bottom of the centrifuge tube. Possible sources of re-contamination were identified by Morrell [30] as follows:

i) insufficient colloid in the tube: the height of the colloid column changes as the buckets assume their horizontal position in the swing-out rotor; thus, unprocessed semen may come into contact with the conical part of the tube where the sperm pellet will eventually be found;
ii) incorrect technique for the removal of the supernatant by having the tip of the extraction pipette below the surface of the supernatant, thus aspirating colloid before all the seminal plasma containing the bacteria has been removed;
iii) accidentally mixing the layers as the tubes are moved from the centrifuge; passing the extraction pipette through contaminated supernatant before reaching the sperm pellet;
iv) lack of access to a laminar air flow bench. Such equipment, although common in laboratories where human semen is processed for fertility treatment, is not usually found at livestock semen collection stations.

**Figure 2:**
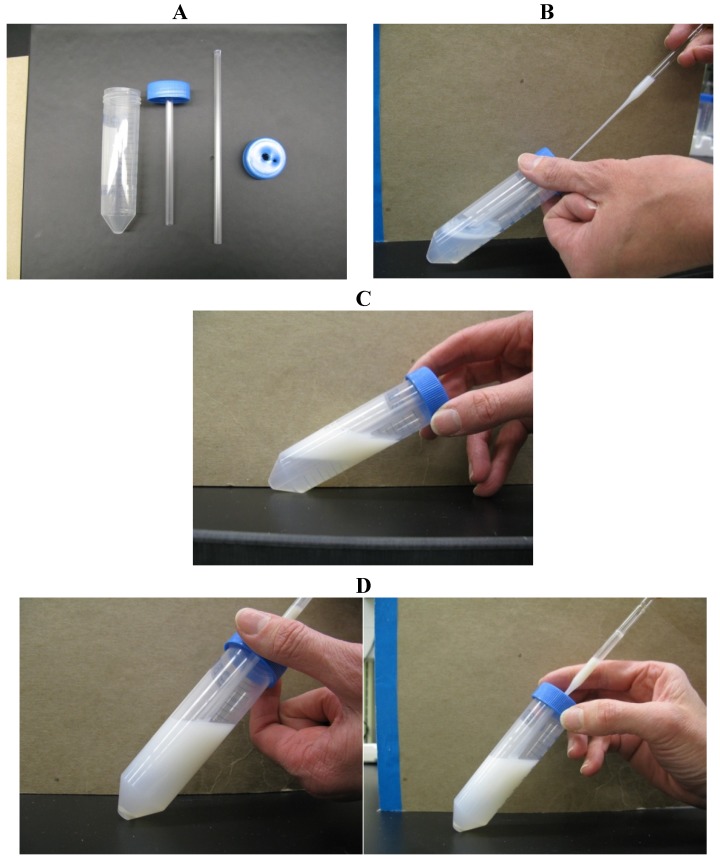
Single-layer centrifugation with a tube insert for retrieval of the sperm pellet. (**A**) Make a hole in the center of the cap for a 50-mL tube, sufficiently large to accommodate a 5-mL plastic semen straw; make a second hole near the perimeter of the cap. (**B**) Pour 15 mL of Androcoll-E Large into the 50-mL tube and screw on the prepared cap. (**C**) Add 15 mL of extended semen via the small hole near the edge of the cap using a Pasteur pipette. Centrifuge the tube at 300× *g* for 20 min in a centrifuge with a swing-out rotor. (**D**) After centrifugation, pass a long Pasteur pipette through the central plastic tube to aspirate the sperm pellet from beneath the colloid.

Note: taken from [34].

To circumvent re-contamination, centrifuge tubes with an insert can be used, to allow removal of the sperm pellet without coming into contact with the supernatant. Such inserts are commercially available for 15-mL centrifuge tubes (Diversified Plastics Inc., Minneapolis, MN, USA) [35] or can be made very easily in the laboratory for other sizes of tube. For example, for 50 mL centrifuge tubes, a 5-mL plastic straw can be passed through a hole punctured in the center of the cap (Figure 2; [34]). Colloid is poured into the open tube, and the cap containing the insemination straw is screwed on in the usual manner. The extended semen is then layered on top of the colloid using a second hole near the periphery of the cap as a port. After centrifugation, a long glass Pasteur pipette is passed slowly down through the central straw to reach the sperm pellet, which can then be aspirated easily without risk of re-contamination from the seminal plasma.

There are two reports of processing semen using tubes inserts, one for human semen [35] and one for stallion semen [25]. Fourie *et al.* [36] reported a study in which semen samples from five men were “spiked” with various bacteria: *Enterobacter cloacae*, *Enterococcus faecalis*, *Escherichia coli*, coagulase-negative staphylococci and *Staphylococcus aureus*, as well as *Candida albicans*, at doses of 1 × 10^3^, 1 × 10^4^, 1 × 10^5^ and 1 × 10^6^ colony forming units/mL (cfu/mL). Although not stated, it is assumed that processing occurred in an LAF bench. The authors reported that significantly more spiking bacteria were removed from the semen samples when processing the semen by DGC using the tube insert than for the controls by DGC without the insert [36]. However, there is no description of the bacterial load in the samples before the spiking bacteria were added, making an exact calculation of the proportion of bacteria removed difficult, if not impossible. Table 3 summarizes their data in terms of the proportion of bacteria remaining after processing by DGC, with or without the tube insert. It would have been interesting if the authors had speculated why certain bacteria were harder to remove by DGC than others, e.g., *Enterococcus faecalis* and coagulase-negative *Staphylococcus*, 40% of which were still present after DGC in at least one sample. Furthermore, 121% of *Enterococcus faecalis* was found in one of the DGC samples, again without explanation.

**Table 3:** Summary of the bacterial load remaining after processing human semen by density gradient centrifugation, with or without a tube insert. DGC, density gradient centrifugation.

Spiking Dose	Method	*Enterobacter-cloacae*	*Enterococcus faecalis*	*Escherichia coli*	Coagulase Neg*. Staphylococcus*	*Staph. aureus*	*Candida albicans*
1 × 10^3^	DGC	10%	0%	10%	10%–40%	0%	0%
1 × 10^4^	DGC	1%–10%	44%–121%	3%–6%	5%–10%	1%	3%–4%
1 × 10^5^	DGC	4%–7%	24%–27%	23%–25%	2%–4%	2%–6%	0.7%–0.9%
1 × 10^6^	DGC	2%–3%	3%–4%	5%–6%	1%–3%	1%–2%	0.2%
1 × 10^3^	DGC + insert	0	0	0	0	0	0
1 × 10^4^	DGC + insert	0	0	0	0	0	0
1 × 10^5^	DGC + insert	0	0.2%	0.1%	0.1%–0.3%	0.4%–0.9%	0
1 × 10^6^	DGC + insert	0.4%–0.7%	6%–10%	0.1%	1%–1.5%	1.5%	0.2%

Note: summarized from [35].

In a similar experiment in 2011 with SLC using a tube insert for stallion semen spiked with *E. coli*, Morrell *et al.* [26] reported the removal of >90% of the total bacterial load (spiking bacteria plus original contaminating bacteria) for bacterial loads of >5 × 10^4^ cfu/mL, although smaller bacterial loads were less efficiently removed. Differences were also seen in the ability of SLC with a tube insert to remove the various species of bacteria found as natural contaminants of the ejaculate, varying from 100% removal for *Enterococcus* to 68% for *Corynebacterium*. These authors suggested that differences in the behavior of the bacteria could inhibit their removal during colloid centrifugation, since the bacteria that were apparently most difficult to remove from stallion spermatozoa (*Corynebacterium* and streptococci) were species that tend to group together, either as palisades or as chains and pairs, respectively, thus potentially altering the density of the bacterial unit as a whole [26].

## 10. Conclusions

To conclude, the addition of antibiotics to semen extenders represents a considerable use of these substances throughout the world, although many microorganisms in semen may be resistant to them. Currently, no other measures are being used to control microbial contamination, although strict attention to hygiene will help to reduce bacterial contamination of semen during collection and processing. Some novel antimicrobials are being studied, but they have not yet appeared on the market. Colloid centrifugation of semen enables a significant proportion of the contaminating bacterial load to be removed, particularly SLC when carried out in an LAF bench using correct aseptic technique. The SLC method has been scaled up to allow voluminous ejaculates (such as from the stallion or boar) to be processed easily at the semen collection station. Variations in the protocol, such as increasing the *g* force for centrifugation, should be avoided, since they may permit more bacteria to pass through the colloid. However, the results suggest that control of bacterial contamination in semen may be possible without the use of antibiotics, although the effects of longer periods of storage and different temperatures of storage need to be investigated for animal semen. Colloid centrifugation should be used as an adjunct to strict attention to hygiene, not as a replacement. As previously mentioned, it is unusual to find LAF benches on semen collection stations, but if serious attempts are to be made to reduce antibiotic usage in the animal breeding industry, the provision of such protective equipment should be considered, together with the adoption of colloid centrifugation as an additional procedure in the semen handling routine.
